# Associations of Less Healthy Snack Food Consumption with Infant Weight-for-Length Z-Score Trajectories: Findings from the Nurture Cohort Study

**DOI:** 10.3390/nu11112752

**Published:** 2019-11-13

**Authors:** Amy M. Moore, Maya Vadiveloo, Alison Tovar, Karen McCurdy, Truls Østbye, Sara E. Benjamin-Neelon

**Affiliations:** 1 Department of Nutrition and Food Sciences, University of Rhode Island, 41 Lower College Rd., Kingston, RI 02881, USA; 2 Department of Human Development and Family Studies, University of Rhode Island, 2 Lower College Rd., Kingston, RI 02881, USA; 3 Department of Family Medicine and Community Health, Duke University Medical Center, DUMC 2914, Durham, NC 27710, USA; 4 Department of Health, Behavior and Society, Johns Hopkins Bloomberg School of Public Health, 615 N. Wolfe St., Baltimore, MD 21205, USA

**Keywords:** infant snacking, less healthy snack foods, infant weight-for-length, Nurture study

## Abstract

Little is known about the impact of less healthy snack foods on weight trajectories during infancy. This secondary analysis of data from the Nurture cohort explored prospective associations of less healthy snack foods with infant weight trajectories. Pregnant women were recruited and, upon delivery of a single live infant, 666 mothers agreed to participate. Mothers completed sociodemographic and infant feeding questionnaires, and infant anthropometrics were collected during home visits at 3, 6, 9, and 12 months. Less healthy snack food consumption was assessed by asking how frequently baby snacks and sweets were consumed each day during the previous three months. Multilevel growth curve models explored associations of baby snacks and sweets with infant weight-for-length (WFL) z-scores. On average, mothers were 27 years old, 71.5% were non-Hispanic Black, and 55.4% had household incomes of ≤$20,000/year. Consumption of less healthy snack foods increased during infancy with a median intake of 3.0 baby snacks/day and 0.7 sweets/day between 10 and 12 months. Growth curve models showed that infants who consumed sweets >2 times/day had significantly higher WFL z-scores during the second half of infancy compared to infants who never consumed sweets. Less healthy snacks may contribute to the risk of obesity during infancy and promoting healthy snack food choices during this critical time is important.

## 1. Introduction

Food preferences and dietary patterns that impact weight trajectories emerge during infancy [1]. Recent national data suggest that 8% of infants and toddlers are at risk for obesity (weight-for-length (WFL) ≥95th percentile) with non-Hispanic Black infants and toddlers at greater risk compared to non-Hispanic Whites [2]. Foods and beverages consumed during infancy influence food preferences and subsequent dietary patterns [3]. For example, higher consumption of fruits and vegetables [4], sweet desserts [3], and sugar-sweetened beverages (SSB) [5] are associated with higher consumption in later childhood. Less healthy dietary patterns, which include foods high in added sugars, sodium, and saturated fats, are associated with an increased risk for obesity during infancy [6] and later childhood [7]. However, there is little evidence exploring the impact of the frequency and timing of less healthy snack foods on infant weight trajectories. There is, therefore, a need to examine the impact of how the frequency and timing of less healthy snack food consumption may contribute to obesogenic dietary patterns. This information will help inform national dietary recommendations for this age group [8,9]. 

Infancy includes a period of rapid dietary transition, from an exclusively milk-based diet to one that includes solid foods [1,10,11]. Parental feeding decisions that follow recommended guidelines, including the appropriate timing and introduction of nutrient-dense solid foods, have been shown to be protective against obesity [12,13,14]. The American Academy of Pediatrics (AAP) recommends the introduction of solid foods at approximately 6 months of age to complement breastmilk or formula [14]. Parents are encouraged to offer a wide variety of healthy foods with varying flavors and textures. Around 9 months of age, meal and snack routines are recommended with three nutrient-dense meals and two or three snacks per day. Parents are also encouraged to avoid less healthy foods with added sugars and limit sodium, saturated fats, and refined grains [14,15]. Despite widespread recognition of the immediate [14,16] and longer-term [17,18] benefits of consuming healthy foods starting early in life, recent National Health and Nutrition Examination Survey (NHANES) data suggest that between 6 and 11 months of age, 25% of infants did not consume any vegetables and 17% did not consume any fruits on a given day [19]. Along with suboptimal vegetable and fruit intake, infants between 6 and 11 months of age consumed a variety of less healthy snack foods with over 50% consuming a sweet or salty snack each day [19]. Similarly, results from the Feeding Infants and Toddlers Study (FITS), a predominantly non-Hispanic White sample, suggest that less healthy snack food consumption increases across infancy, with 9% of infants between 6 and 8 months of age and nearly 20% of infants between 9 and 11 months of age consuming at least one sweet or salty snack each day [20]. Disparities also begin to emerge during infancy with non-Hispanic Black infants consuming fewer vegetables and fruits and more sweet and salty snacks compared to non-Hispanic White infants [19]. Increasing trends in the consumption of less healthy snacks during infancy is particularly concerning given these snacks may displace healthier foods and also contribute excess calories. Additionally, there is limited research on less healthy snacking in low-income, non-Hispanic infants, which makes examining snacking in this population important. 

Recent increases in childhood obesity coincide with increases in snack food marketing, snacking frequency, and overall calorie contribution from snack foods [21,22,23], with the greatest increases in populations most at risk for childhood obesity, including low-income and non-Hispanic Black households [20,24]. Experts agree that the impact of snack foods on weight status depends on the frequency and energy-density of snack foods [22,24]. Preschool-aged children consume approximately three snacks per day, which contribute nearly 30% of daily calories with the majority of those calories coming from less healthy snacks [19,22,23]. This increases during early childhood with nearly 54% of daily calories coming from less healthy snacks [23]. However, studies with older children and adolescents reveal that snacking may have favorable effects on weight status due to increases in healthy snack foods (e.g., fruits and vegetables) [25,26]. In contrast to older children, recent studies of preschool-aged children suggest that a large proportion of snack foods are from less healthy sweet and salty snacks, and these energy-dense snack foods may lead to excess calories and subsequent obesity [22,23,27]. However, little is known about the impact of less healthy snack foods on weight trajectories during the first year of life. Therefore, the purpose of this analysis was to: 1) describe the prevalence of less healthy snack food (baby snacks and sweets) consumption across important transitions in infant feeding, and 2) explore the relationship of baby snacks and sweets with infant WFL z-score trajectories in a predominantly low-income, racially diverse cohort. We hypothesized that greater consumption of baby snacks and sweets would be associated with higher WFL z-scores after controlling for confounding variables. This study is one of the first to examine the impact of less healthy snack foods on infant weight trajectories in a low-income, diverse cohort.

## 2. Materials and Methods 

### 2.1. Study Design and Participants

This was a secondary analysis of data from the Nurture study, a prospective observational birth cohort of predominantly non-Hispanic Black mothers and their infants residing in the Southeastern USA [28]. The Nurture study was designed to explore longitudinal associations between various infant caregivers and infant adiposity during the first year of life. Women between 20 and 36 weeks’ gestation were recruited from a county health department prenatal clinic and a private prenatal clinic in Durham, North Carolina from 2013 to 2015. Recruited mothers were ≥18 years of age with a singleton pregnancy with no known congenital abnormalities. After delivery, mothers confirmed continued interest in participating or were excluded if: no longer interested, their infants were born before 37 weeks gestation or were unable to take breastmilk or formula by mouth at hospital discharge. A total of 666 mother-infant dyads were enrolled in the study. Details on the study design have been provided elsewhere [28]. Mothers provided written informed consent and parental permission for their infants. All procedures were approved by Duke University Medical Center Institutional Review Board (human subjects committee, Pro 0036242). 

### 2.2. Measures

Data collection occurred from 2013 to 2016. Trained data collectors conducted four home visits when infants were 3, 6, 9, and 12 months of age. Mothers completed sociodemographic and infant feeding questionnaires, and infant heights and weights were measured during each home visit. Mothers reported how frequently infants consumed foods and beverages each day during the previous three months using items from the Infant Feeding Practices Study II (IFPS II) [29] and the Feeding Infants and Toddlers Study (FITS) [30]. Items included how frequently infants consumed baby snacks (teething biscuits, puffs, and melts), sweets (cookies, cakes, or candy), SSBs, fruits (not including fruit juice), vegetables (not including vegetable juice), dairy (yogurt and cheese), protein (meat, fish, and eggs), grains (breakfast cereals, crackers, bread, pasta, and rice), breastmilk, and infant formula each day. Based on AAP recommendations for the introduction of solid foods [14], any foods and beverages (other than breastmilk and infant formula) consumed between birth and 3 months of age were further categorized as early introduction to solid foods.

#### 2.2.1. Sociodemographics

Sociodemographic characteristics were collected at recruitment and during each home visit. Maternal variables of interest included age, pre-pregnancy body mass index (BMI), race (black, white, or other), education (≤high school diploma, some college, college graduate, or graduate degree), household income (≤$20,000, $20,001–$40,000, ≥$40,001), Special Supplemental Nutrition Program for Women, Infants, and Children (WIC) status, and total number of weeks of any breastfeeding between birth and 12 months. Infant variables of interest included gender, birth weight for gestational age (WGA) z-scores, and WFL z-scores. 

#### 2.2.2. Exposure Variable (Categorical)—Baby Snacks and Sweets

Less healthy snack food consumption during the first year of life was assessed using two items: 1) “How often was he or she (their infant) fed baby snacks (teething biscuits, puffs, or melts) during the month” and 2) “How often was he or she [their infant] fed sweets (cookies, cakes, or candy) during the month”. Response options for both items were: 0 = never, 1 = just to try, 2 = sometimes but less than once/day, and from 3 = 1 time/day to 7 = 5 or more times/day. To capture important transitions in infant feeding, monthly responses for baby snacks and sweets were averaged across three months (4–6 months, 7–9 months, and 10–12 months) to create an average score for each time point. To reflect AAP recommendations for snacking frequency, averaged scores for baby snacks were further categorized as: 0 = never (never, just to try, or less than once/day), 1 = sometimes (1–3 times/day), or 2 = often (>3 times/day). Given the narrow distribution of scores for sweets, averaged scores were categorized as: 0 = never (never, just to try, or less than once/day), 1 = sometimes (1–2 times/day), or 2 = often (>2 times/day). 

#### 2.2.3. Outcome Variable (Continuous)—Infant WFL z-Scores

Standardized measurements of infant recumbent length (ShorrBoard Portable Length Board, Issaquah, WA) and weight (Seca Infant Scale, Hanover, MD) were collected in triplicate by trained staff during the four home visits. An average of the three measurements was used to calculate age- and sex-specific WFL z-scores at 3, 6, 9, and 12 months using World Health Organization reference standards [31].

#### 2.2.4. Covariates

Several mother and infant sociodemographic characteristics were examined as possible covariates based on previous findings of an association with infant weight status (pre-pregnancy BMI, birth WGA z-scores, total number of weeks any breastfeeding (non-exclusive), and early introduction of solid foods) [17]. Other possible covariates were examined based on research suggesting an association with infant feeding and infant weight status (mother’s age, race, education, household income, and infant gender) [29]. Covariates were included in adjusted models when the magnitude of the association between baby snacks or sweets consumed and WFL z-scores changed by approximately 10% when added separately to the model [32]. 

### 2.3. Statistical Analyses

Descriptive statistics including means and standard deviations (SD) or medians and interquartile ranges (IQR) for continuous variables and frequencies and percentages for categorical variables were used to summarize sociodemographic characteristics and infant feeding. Spearman’s rho correlation coefficients were computed to examine the associations between foods and SSBs (as continuous variables) consumed averaged across three time points (i.e., 4–12 months). Multilevel growth curve models were used to explore the prospective associations of baby snacks and sweets consumed between 4–6 months, 7–9 months, and 10–12 months with infant WFL z-scores at 6 months, 9 months, and 12 months. These models are appropriate for longitudinal data with repeated measures, and were built following published guidelines [33], with infant age as the measure of time point (level 1) nested within infants (level 2). Baby snacks and sweets were modeled as fixed effects with individual infants’ slopes and intercepts modeled as random effects. Models were estimated using restricted maximum likelihood estimation (REML) [33], and a change in Bayesian Information Criteria (BIC) of >10 was used to indicate significant improvement in model fit [34]. All participants who completed at least one home visit were included in models. First, an unconditional means model (model 1) with no predictors was estimated and used to calculate the intraclass correlation coefficient (ICC). A larger ICC indicates more between-infant variation and a smaller ICC indicates more within-infant variation in WFL z-scores. Second, an unconditional (unadjusted) growth model (model 2) was estimated to examine the impact of time point as a fixed effect on infant WFL z-scores. Time point was centered at 4–6 months to reflect recommendations for solid food introduction, such that the intercept represented mean WFL z-scores at 6 months, and the slope represented change in mean WFL z-scores per time point. Next, conditional (adjusted) growth models were estimated to examine the effects of level 2 categorical predictors, baby snacks (model 3) and sweets (model 4). Baby snacks and sweets were modeled as never (reference), sometimes, and often to examine differences in less healthy snack food frequency and timing on WFL z-scores. Both models were adjusted to control for the potential confounding effects of infant birth WGA z-scores and total number of weeks of any breastfeeding (i.e., the only covariates changing the magnitude of the association by approximately 10%). Interactions between baby snacks and sweets and maternal covariates (race, pre-pregnancy BMI, and income) were explored and were not significant at *p* < 0.05. All analyses were conducted using SAS 9.4 (SAS Institute, Cary, NC, USA), and a *p* value of < 0.05 was used to determine statistical significance. 

## 3. Results

### 3.1. Sociodemographics

Table 1 shows sociodemographic characteristics of mother-infant dyads participating in the Nurture study. Mothers were on average 27.1 (SD = 5.8) years of age with a mean pre-pregnancy BMI of 29.9 (SD = 9.3). Mothers were predominantly non-Hispanic Black (71.5%) with over half reporting a household income of ≤$20,000/year (55.4%). Nearly half of infants were female (48.8%), and birth WGA z-scores (*M* = −0.3, SD = 0.9) and WFL z-scores (across all time points) were within the normal range. Infants were non-exclusively breastfed an average of 14.7 weeks (SD = 18.2) with 82.9% of infants consuming breastmilk at least one time per day between birth-3 months of age, 54.7% between 4–6 months, 39.3% between 7–9 months, and 35.2% between 10–12 months. One-third of mothers (30.3%) reported introducing any solid foods before 4 months of age. 

### 3.2. Consumption of Baby Snacks and Sweets during the First Year of Life

Very few mothers introduced baby snacks (*n* = 7) or sweets (*n* = 3) between birth and 3 months. Overall, 25.8% of infants consumed at least one baby snack per day between 4–6 months, 82.2% between 7–9 months, and 87.6% between 10–12 months. Similarly, 7.1% of infants consumed at least one sweet per day between 4–6 months, 28.7% between 7–9 months, and 47.2% between 10–12 months. Table 2 shows medians (IQR) for baby snacks and sweets consumed per day across the first year of life. Baby snacks consumption increased across the first year of life reaching a median of 3.0 (IQR = 2.0–4.0) times/day between 10–12 months. Sweets consumption also increased reaching a median of 0.7 (IQR = 0.0–1.7) times/day between 10–12 months.

Spearman’s rho correlations for less healthy snacks and other foods consumed from 4 to 12 months are shown in Table S1. There was a weak negative correlation between baby snacks (*ρ* = −0.16) and sweets (*ρ* = −0.12) with breastmilk, and a weak positive correlation between baby snacks (*ρ* = 0.03) and sweets (*ρ* = 0.06) and infant formula. Baby snacks were positively correlated with all other foods and SSBs with values ranging from 0.17 to 0.47. Similarly, sweets were positively correlated with all other foods and SSBs with values ranging from 0.22 to 0.49.

### 3.3. Association of Baby Snacks and Sweets with Infant WFL Z-Scores Trajectories

**Figure 1:**
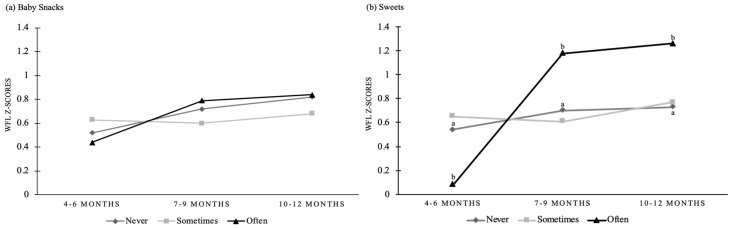
Adjusted models for change in (**a**) baby snacks and (**b**) sweets consumption and weight-for-length (WFL) z-scores by time point for infants in the Nurture Study. Baby snacks and sweets consumption are categorized as never, sometimes or often. a–b, significant sweets x time interaction for infants consuming snacks often when compared to never, *p* < 0.01. There were no other significant interactions.

Multilevel growth curve models for prospective associations of baby snacks and sweets consumed at 4–6 months, 7−9 months, and 10−12 months with infant WFL z-scores at 6 months, 9 months, and 12 months are shown in Table 3 and Figure 1. A mean WFL z-score of β = 0.52 (SE = 0.04, *p* < 0.001) was observed for the unconditional means model (model 1), which assumes static WFL z-scores across infancy. Model 1 also shows that 79% of the variance (ICC 0.79) in infant WFL z-score exists between infants. The unconditional (unadjusted) growth model (Model 2), shows that infant WFL z-score trajectories increased and were within the normal range across the time points (4−6 months, β = 0.38, SE = 0.05, *p* < 0.001; 7−9 months, β = 0.17, SE = 0.03, *p* < 0.001; 10−12 months, β = 0.25, SE = 0.03, *p* < 0.001). In conditional (adjusted) growth models that included baby snacks (model 3), infant WFL z-score trajectories were on average higher at 4–6 months compared to unconditional growth models and increased across the time points (4−6 months, β = 0.52, SE = 0.06, *p* < 0.001; 7−9 months, β = 0.20, SE = 0.07, *p* = 0.005; 10−12 months, β = 0.30, SE = 0.08, *p* < 0.001). However, there were no significant main or interaction effects of baby snacks with infant WFL z-score trajectories for any category (never, sometimes, or often) or time point (4−6, 7−9, or 10−12 months). In conditional (adjusted) growth models that included sweets (model 4), infant WFL z-score trajectories were on average higher at 4−6 months when compared to unconditional growth models and increased across the time points (4−6 months, β = 0.54, SE = 0.06, *p* < 0.001; 7−9 months, β = 0.16, SE = 0.04, *p* < 0.001; 10−12 months, β = 0.19, SE = 0.04, *p* < 0.001). The main effect of sweets (F_2246_ = 3.23, *p* = 0.03) and interaction effects of sweets by time point (F_4805_ = 2.44, *p* = 0.04) on WFL z-score trajectories were significant. There was a significant sweets by time point interaction at 4−6 months, infants consuming sweets often was negatively associated with infant WFL z-scores (β = −0.46, SE = 0.17, *p* = 0.009) when compared to infants who never consumed snacks. There was a significant sweets by time point interaction at 7−9 months, infants consuming sweets often was positively associated with infant WFL z-scores (β = 0.48, SE = 0.18, *p* = 0.01) when compared with infants who never had sweets. There was also a significant sweets by time point interaction at 10−12 months, infants consuming sweets often was positively associated with infant WFL z-scores (β = 0.53, SE = 0.18, *p* = 0.004) when compared to infants who never had sweets. There were no other significant sweets by time point interactions. 

## 4. Discussion

The goal of this analysis was to explore the association between less healthy snack foods (baby snacks and sweets) and infant WFL z-score trajectories during the first year of life. In this sample of predominantly low-income, non-Hispanic Black mothers and their infants, 25% of infants consumed baby snacks and 7% consumed sweets between 4–6 months of age. There was an increasing trend across the first year of life, where 87% of infants consumed baby snacks and 47% consumed sweets by 10−12 months of age. Our results suggest that consuming sweets impacts infant weight trajectories. At 7−9 and 10−12 months of age, infants consuming sweets often (>2 times /day) had higher WFL z-scores compared to infants who never had sweets. Continued efforts to reduce less healthy foods during infancy, especially sweets (e.g., cookies, cakes, and candies), may be critical to the development of healthy food preferences, dietary patterns, and weight trajectories that begin to emerge during this early developmental period.

The findings that mothers introduced less healthy snack foods during the first year of life are consistent with previous research [19,20]. Data from NHANES found increasing trends for less healthy snack foods, where 5% of young infants (birth–5 months) and 50% of older infants (6−11 months) consumed a sweet or salty snack each day, which is consistent with the IFPS II and our study. The AAP recommends three nutrient-dense small meals and two or three small snacks per day [14], which leaves little room for discretionary calories from nutrient-poor foods. Notably, the FITS found that self-reported snack foods provided about one-fifth of an infant’s daily energy needs [35]. Although not all snacks were nutrient-poor (e.g., 48% consumed fruits and 9% consumed vegetables), over 20% of infants consumed sweets, SSBs, or desserts. Given the prevalence and energy contribution of less healthy snack foods during the first year of life, it is critical to examine if these snacking patterns contribute to early weight gain and to investigate factors that influence parents and other caregivers to offer less healthy snack foods during infancy. 

This study adds to the literature as one of the first to explore the association between less healthy snack foods with infant WFL z-score trajectories. In line with our a priori hypothesis, sweet consumption had a significant impact on weight trajectories between 4–12 months of age. Of interest is our finding that infants who consumed sweets more often (>2 times/day) during early complementary feeding (4−6 months) had lower WFL z-scores. Although this was a significant finding, results should be interpreted with caution given the very small sample size in the sweets category. In contrast, between 7–9 and 10–12 months, infants who consumed sweets often had higher WFL z-scores compared to infants who never consumed sweets. This is also in contrast to other studies finding no association or a protective association between snack foods and weight status in older children [25,26,36]. However, our study targeted less healthy snacking during infancy and results may reflect that infants have little room for discretionary or “empty” calories. In contrast to our a priori hypothesis, baby snacks did not have a significant impact on infant weight trajectories at any category or time point. This finding suggests that commercially available baby snacks may not contribute sufficient discretionary calories that may place infants at risk for obesity. 

To our knowledge, this was one of the first studies to explore the impact of commercially available baby snacks (teething biscuits, puffs, and melts), which are snacks marketed specifically to the parents of infants, on infant weight trajectories. A recent study found that many commercially available infant and toddler foods contain added sugars and salt [37], which are not recommended for this age group [14] and may also contribute excess calories. Although baby snacks did not have a significant impact on infant weight trajectories in our study this area warrants further exploration. Particularly given that food and beverages offered during early infancy influence food preferences, dietary patterns, and weight trajectories that often persist into later childhood, further exploring the impact of commercially available baby snacks on infant weight status and promoting healthy snacking is important. 

The primary limitation of this study was the use of self-report infant dietary questionnaires, which have been shown to be biased by under- and overreporting, to examine less healthy snack food consumption. However, the dietary questionnaire has been used in other cohort studies [29,30], and the validity is supported by similar findings of less healthy snack food consumption in a large national sample [19]. The dietary questionnaire also did not include serving sizes, so the energy contribution of baby snacks and sweets is unknown. Future studies should consider including 24 hour recalls to collect data on serving size in order to assess the energy contribution of snack foods. To assess less healthy snacking behaviors, our study examined baby snacks (teething biscuits, puffs, and melts) and sweets (cookies, cakes, and candies); however, it is unknown if mothers would describe these foods as snacks or if there are other foods that mothers would describe as snacks that were not included in our analysis. However, given recent increases in less healthy snack food consumption during early childhood [22], these snack foods warrant examination as independent predictors of infant weight trajectories. Future studies may consider qualitative research with mothers of young infants to understand how mothers define snacking and to explore factors that influence snacking during infancy. Although social desirability bias may contribute to underreporting of less healthy snack food consumption, the prevalence of less healthy snack foods in our sample were in line with other studies assessing snack food consumption during infancy [19,20]. In addition, very few infants in the Nurture study had WFL z-scores placing them at risk for obesity, therefore we were unable to examine the impact of less healthy snacks by weight status (i.e., normal weight compared to infants at risk for obesity). 

This study adds to the literature on snack food consumption during infancy and has a number of strengths that warrant mention. This analysis included primarily low-income, non-Hispanic Black mothers and their infants who are underrepresented in the research literature. Including underrepresented groups in research is a public health priority and is a vital component of reducing health disparities [38]. In addition, this study examined less healthy snack food consumption across important transitions in infant feeding (i.e., from a milk-based diet to solid foods). Given that less healthy dietary patterns that begin during infancy often track into later childhood and have been associated with increased weight status, examining the impact less healthy snacks on weight during infancy is essential. In addition, future studies may consider examining snack food consumption and weight status during the toddler years to understand how snacking and weight trajectories track across the first two years of life. This study also highlights the need for recommendations around healthy snack foods, particularly in this young age group who are learning to eat and developing food preferences. 

## 5. Conclusions

This analysis of the Nurture study, a cohort of predominantly low-income, non-Hispanic Black mothers and their infants, found that mothers introduce less healthy snacks during the first year of life. Our results suggest that less healthy sweets (cookies, cakes, and candies) are associated with increased weight trajectories during later infancy, making these snack foods important targets for childhood obesity prevention efforts. Given that less healthy snacks are offered during early infancy and may contribute to the risk of adiposity, promoting healthy snack food choices during this critical window is important. Future studies should explore drivers of snacking during infancy to help inform the evidence-base for healthy snacking recommendation during infancy. In addition, understanding the drivers of infant snacking would help to inform developmentally appropriate infant feeding interventions and help to address factors in the food environment that influence less healthy snacking during infancy.

## Figures and Tables

**Table 1:** Sociodemographic characteristics of mother-infant dyads participating in the Nurture study.

**Mother Characteristics**	***n***	**Mean (SD) or %**
Age (years)	666	27.1 (5.8)
Pregnancy BMI	666	29.9 (9.3)
Race	661	
Black		71.5
White		19.2
Other		8.9
Ethnicity, Latina	661	6.5
Education	663	
≤High school diploma		47.8
Some college		30.3
College graduate		15.5
Graduate degree		6.3
Household Income	607	
≤$20,000		55.4
$2,0001–$4,0000		19.1
≥$4,0001		16.7
**Infant Characteristics**	***n***	**Mean (SD) or %**
Gender, female	666	48.8
Race	661	
Black		68.6
White		15.0
Other		14.9
Ethnicity, Latina/o	661	8.9
Birth WGA z-score	666	−0.31 (0.9)
WFL z-score, mean		
3 months	534	0.14 (1.1)
6 months	492	0.39 (1.1)
9 months	456	0.56 (1.0)
12 months	466	0.64 (1.0)
Total Weeks Breastfed ^a^	657	14.7 (18.2)
Early Introduction of Solids ^b^	534	30.3

^a^ Includes any breast feeding. ^b^ Includes any foods and SSB (other than breastmilk or formula), consumed from birth-3 months of age. WGA, weight-for-gestational age; WFL, weight-for-length.

**Table 2:** Medians (interquartile range, IQR) for selected foods and sugar-sweetened beverages consumed by infants per day.

Infant Dietary Characteristics	0 to 3 Months	4 to 6 Months	7 to 9 Months	10 to 12 Months
	*n* = 534	*n* = 492	*n* = 456	*n* = 466
Baby snacks ^a^	0.0 (0.0–0.0)	0.0 (0.0–1.0)	2.7 (1.3–3.7)	3.0 (2.0–4.0)
Sweets ^b^	0.0 (0.0–0.0)	0.0 (0.0–0.0)	0.0 (0.0–1.0)	0.7 (0.0–1.7)
SSB ^c^	0.0 (0.0–0.0)	0.0 (0.0–0.0)	0.0 (0.0–1.7)	1.0 (0.0–2.7)
Fruits	0.0 (0.0–0.0)	0.7 (0.0–2.0)	3.0 (2.0–4.0)	3.7 (2.7–4.0)
Vegetables	0.0 (0.0–0.0)	0.7 (0.0–2.3)	3.0 (2.0–4.0)	3.7 (2.7–4.0)
Breastmilk	5.0 (2.3–7.0)	4.7 (0.0–7.0)	6.0 (0.0–7.0)	4.2 (0.0–7.0)
Formula	5.3 (2.0–7.0)	6.7 (5.0–7.0)	6.0 (5.0–7.0)	5.3 (3.3–6.0)

^a^ Includes teething biscuits, puffs, and melts. ^b^ Includes cakes, cookies, and candies. ^c^ SSB (sugar-sweetened beverages) includes juice drinks, soda, and sweetened tea.

**Table 3:** Results for multilevel growth curve models for infant WFL z-score trajectories.

		Model 1 Unconditional Means	Model 2 Unconditional Growth	Model 3 Conditional Growth	Model 4 Conditional Growth
	*n*	*n* = 666	*n* = 666	*n* = 532	*n* = 532
Fixed Effects					
Intercept					
Initial Status (Time_1_)		0.52 (0.04) **	0.38 (0.05) **	0.52 (0.06) **	0.54 (0.06) **
Slope (change in WFL z-scores)					
Time_1_			0	0	0
Time_2_			0.17 (0.03) **	0.20 (0.07) **	0.16 (0.04) **
Time_3_			0.25 (0.03) **	0.30 (0.08) **	0.19 (0.04) **
Baby Snacks × Time_1_					
Never	364			0	
Sometimes	91			0.11 (0.07)	
Often	36			−0.08 (0.10)	
Baby Snacks × Time_2_					
Never	76			0	
Sometimes	174			−0.12 (0.11)	
Often	201			0.07 (0.12)	
Baby Snacks × Time_3_					
Never	57			0	
Sometimes	132			−0.14 (0.11)	
Often	276			0.02 (0.13)	
Sweets × Time_1_					
Never	456				.0
Sometimes	24				0.11 (0.12)
Often	11				−0.46 (0.17) *
Sweets × Time_2_					
Never	320				.0
Sometimes	66				−0.09 (0.14)
Often	65				0.48 (0.18) *
Sweets × Time_3_					
Never	245				.0
Sometimes	109				0.04 (0.14)
Often	111				0.53 (0.18) *
Random Effects					
Level 1		0.22 (0.01) **	0.21 (0.01) **	0.20 (0.01) **	0.20 (0.05) **
Intercept		0.84 (0.06) **	0.85 (0.06) **	0.79 (0.05) **	0.79 (0.05) **
Model Fit					
BIC		3023.7	2978.4	2926.3	2919.0

Table 3 includes parameter estimates with standard errors in parentheses. Models 3 (baby snacks) and 4 (sweets) adjusted for birth weight-for-gestational age z-scores and total weeks any breastfeeding. Baby snacks and sweets were modeled as never [reference], sometimes, and often. Time was modeled as infant age (time_1_ = 4–6 months [reference]; time_2_ = 7−9 months; time_3_ = 10−12 months). Covariance Structure = VC; Estimation Method = REML; Between-within degrees of freedom. Model 1 ICC = 0.79; * *p* < 0.01, ** *p* < 0.001.

## Supplementary Materials

The following are available online at https://www.mdpi.com/2072-6643/11/11/2752/s1, Table S1: Bivariate correlations between foods and sugar-sweetened beverages (SSB) consumed by infants from 4 to 12 months.

## References

[1] Birch L.L., Doub A.E. (2014). Learning to eat: Birth to age 2 y. Am. J. Clin. Nutr..

[2] Ogden C.L., Carroll M.D., Kit B.K., Flegal M. (2014). Prevalence of childhood and adult obesity in the United States, 2011–2012. JAMA.

[3] Rose C.M., Birch L.L., Savage J.S. (2017). Dietary patterns in infancy are associated with child diet and weight outcomes at 6 years. Int. J. Obes..

[4] Saavedra J.M., Deming D., Dattilo A., Reidy K. (2013). Lessons from the Feeding Infants and Toddlers Study in North America: What children eat, and implications for obesity prevention. Ann. Nutr. Metab..

[5] Park S., Pan L., Sherry B., Li R. (2014). The association of sugar-sweetened beverage intake during infancy with sugar-sweetened beverage intake at 6 years of age. Pediatrics.

[6] Vadiveloo M., Tovar A., Østbye T., Benjamin-Neelon S.E. (2019). Associations between timing and quality of solid food introduction with infant weight-for-length z-scores at 12 months: Findings from the Nurture cohort. Appetite.

[7] Pan L., Li R., Park S., Galuska D.A., Sherry B., Freedman D.S. (2014). A longitudinal analysis of sugar-sweetened beverage intake in infancy and obesity at 6 years. Pediatrics.

[8] English L.K., Obbagy J.E., Wong Y.P., Butte N.F., Dewey K.G., Fox M.K., Greer F.R., Krebs N.F., Scanlon K.S., Stoody E.E. (2019). Timing of introduction of complementary foods and beverages and growth, size, and body composition: A systematic review. Am. J. Clin. Nutr..

[9] English L.K., Obbagy J.E., Wong Y.P., Butte N.F., Dewey K.G., Fox M.K., Greer F.R., Krebs N.F., Scanlon K.S., Stoody E.E. (2019). Types and amounts of complementary foods and beverages and growth, size, and body composition: A systematic review. Am. J. Clin. Nutr..

[10] Savage J., Fisher J., Birch L. (2007). Parental influences on eating behavior: Conception to adolescence. J. Law Med. Ethics.

[11] Birch L., Savage J.S., Ventura A. (2007). Influences on the development of children’s eating behaviours: From infancy to adolescence. Can. J. Diet. Pract. Res..

[12] Woo Baidal J.A., Locks L.M., Cheng E.R., Blake-Lamb T.L., Perkins M.E., Taveras E.M. (2016). Risk factors for childhood obesity in the first 1,000 days. Am. J. Prev. Med..

[13] Wang J., Wu Y., Xiong G., Chao T., Jin Q., Liu R., Hao L., Wei S., Yang N., Yang X. (2016). Introduction of complementary feeding before 4 months of age increases the risk of childhood overweight or obesity: A meta-analysis of prospective cohort studies. Nutr. Res..

[14] Kleinman R.E., Greer F.R. (2014). Pediatric Nutrition.

[15] Vos M.B., Kaar J.L., Welsh J.A., Van Horn L.V., Feig D.I., Anderson C.A.M., Patel M.J., Cruz Munos J., Krebs N.F., Xanthakos S.A. (2016). Added sugars and cardiovascular disease risk in children—A scientific statement from the American Heart Association. Circulation.

[16] Young B.E., Krebs N.F. (2014). Complementary feeding: Critical considerations to optimize growth, nutrition, and feeding behavior. Curr. Pediatr. Rep..

[17] Weng S.F., Redsell S.A., Swift J.A., Yang M., Glazebrook C.P. (2012). Systematic review and meta-analyses of risk factors for childhood overweight identifiable during infancy. Arch. Dis. Child..

[18] Pearce J., Taylor M.A., Langley-Evans S.C. (2013). Timing of the introduction of complementary feeding and risk of childhood obesity: A systematic review. Int. J. Obes..

[19] Miles G., Siega-Riz A.M. (2017). Trends in food and beverage consumption among infants and toddlers: 2005–2012. Pediatrics.

[20] Deming D.M., Reidy K.C., Fox M.K., Briefel R.R., Jacquier E., Eldridge A.L. (2017). Cross-sectional analysis of eating patterns and snacking in the US Feeding Infants and Toddlers Study 2008. Public Health Nutr..

[21] Harris J.L., Fleming-Milici F., Frazier W., Haraghey K., Rodriguez-Arauz G., Heller R., Hubbard W. (2017). Baby Food FACTS: Nutrition and Marketing of Baby and Toddler Food and Drinks.

[22] Piernas C., Popkin B.M. (2010). Trends in snacking among U.S. children. Health Aff..

[23] Dunford E.K., Popkin B.M. (2018). 37 year snacking trends for US children 1977–2014. Pediatr. Obes..

[24] Charvet A., Hartlieb K.B., Yeh Y., Jen K.-L.C. (2016). A comparison of snack serving sizes to USDA guidelines in healthy weight and overweight minority preschool children enrolled in Head Start. BMC Obes..

[25] Evans W.E., Jacques P.F., Dallal G.E., Sacheck J., Must A. (2015). The role of eating frequency on total energy intake and diet quality in a low-income, racially diverse sample of schoolchildren. Public Health Nutr..

[26] Keast D.R., Nicklas T.A., O’Neil C.E. (2010). Snacking is associated with reduced risk of overweight and reduced abdominal obesity in adolescents: National Health and Nutrition Examination Survey (NHANES) 1999–2004. Am. J. Clin. Nutr..

[27] Shriver L.H., Marriage B.J., Bloch T.D., Spees C.K., Ramsay S.A., Watowicz R.P., Taylor C.A. (2018). Contribution of snacks to dietary intakes of young children in the United States. Matern. Child Nutr..

[28] Neelon S.E.B., Østbye T., Bennett G.G., Kravitz R.M., Clancy S.M., Stroo M., Iversen E., Hoyo C. (2017). Cohort profile for the Nurture Observational Study examining associations of multiple caregivers on infant growth in the Southeastern USA. BMJ Open.

[29] Fein S.B., Labiner-Wolfe J., Shealy K.R., Li R., Chen J., Grummer-Strawn L.M. (2008). Infant Feeding Practices Study II: Study methods. Pediatrics.

[30] Briefel R.R., Kalb L.M., Condon E., Deming D.M., Clusen N.A., Fox M.K., Harnack L., Gemmill E., Stevens M., Reidy K.C. (2010). The Feeding Infants and Toddlers Study 2008: Study design and methods. J. Am. Diet. Assoc..

[31] World Health Organization (2006). Child Growth Standards: Length/Height-for-Age, Weight-for-Age, Weight-for-Length, Weight-for-Height and Body Mass Index-for-Age: Methods and Development.

[32] Maldonado G., Greenland S. (1993). Simulation study of confounder-selection strategies. Am. J. Epidemiol..

[33] Singer J.D., Willett J.B. (2009). Applied Longitudinal Data Analysis: Modeling Change and Event Occurrence.

[34] Raftery A.E. (1995). Bayesian model selection in social research. Sociol. Methodol..

[35] Jacquier E.F., Deming D.M., Eldridge A.L. (2018). Location influences snacking behavior of US infants, toddlers and preschool children. BMC Public Health.

[36] Larson N., Story M. (2013). A review of snacking patterns among children and adolescents: What are the implications of snacking for weight status?. Child. Obes..

[37] Maalouf J., Cogswell M.E., Bates M., Yuan K., Scanlon K.S., Pehrsson P., Gunn J.P., Merritt R.K. (2017). Sodium, sugar, and fat content of complementary infant and toddler foods sold in the United States, 2015. Am. J. Clin. Nutr..

[38] Erves J.C., Mayo-Gamble T.L., Malin-Fair A., Boyer A., Joosten Y., Vaughn Y.C., Sherden L., Luther P., Miller S., Wilkins C.H. (2017). Needs, priorities, and recommendations for engaging underrepresented populations in clinical research: A community perspective. J. Community Health.

