# Supplementary material for: Associations of Less Healthy Snack Food Consumption with Infant Weight-for-Length Z-Score Trajectories: Findings from the Nurture Cohort Study
Source: Nutrients. 2019 Nov 13;11(11):2752. doi: 10.3390/nu11112752 (PMC6893797; doi:10.3390/nu11112752)
Supplement: Supplementary file 1 [file nutrients-11-02752-s001.pdf]

nutrients MDPI

Table S1. Bivariate correlations between foods and sugar-sweetened beverages (SSB) consumed by infants from 4 to 12 months.

	Baby Snacks [a]	Sweets [b]	SSB [c]	Fruits	Vegetables	Dairy [d]	Protein [e]	Grains [f]	Breastmilk	Formula
Baby snacks [a]	–									
Sweets [b]	0.30 *	–								
SSB [c]	0.17 **	0.48 *	–							
Fruits	0.43 *	0.23 *	0.07	–						
Vegetables	0.41 *	0.22 *	0.13 **	0.78 *	–					
Dairy [d]	0.39 *	0.37 *	0.24 *	0.42 *	0.44 *	–				
Protein [e]	0.39 *	0.49 *	0.29 *	0.43 *	0.47 *	0.53 *	–			
Grains [f]	0.47 *	0.31 *	0.27 *	0.35 **	0.40 *	0.29 *	0.37 *	–		
Breastmilk	-0.16 **	−0.12 **	−0.27 *	0.01	0.04	0.08	−0.04	−0.26 *	–	
Formula	0.03	0.06	0.08 *	−0.06	−0.05	−0.17 *	−0.18 *	0.15 **	−0.63 *	–

[a] Includes teething biscuits, puffs, and melts. [b] Includes cakes, cookies, and candies. [c] Includes juice drinks, soda, or sweeten tea. [d] Includes yogurt and cheese. [e] Includes meat, fish and eggs. [f] Includes breakfast cereal, crackers, bread, pasta, rice, and infant cereal. ** $p < 0.05$, * $p < 0.001$
